# Synthesis of No-Carrier-Added 4-[^18^F]Fluorophenol from 4-Benzyloxyphenyl-(2-thienyl)iodonium Bromide

**DOI:** 10.3390/molecules16097621

**Published:** 2011-09-06

**Authors:** Tobias L. Ross, Johannes Ermert, Heinz H. Coenen

**Affiliations:** Institute of Neuroscience and Medicine, INM-5: Nuclear Chemistry, Forschungszentrum Jülich GmbH, 52425 Jülich, Germany

**Keywords:** fluorine-18, 4-[^18^F]fluorophenol, diaryl iodonium salts, radiosynthesis, positron emission tomography

## Abstract

4-[^18^F]Fluorophenol is a versatile synthon for the synthesis of more complex radiopharmaceuticals bearing a 4-[^18^F]fluorophenoxy moiety. In order to prepare 4-[^18^F]fluorophenol in no-carrier-added (n.c.a.) form only a nucleophilic labelling method starting from [^18^F]fluoride is suitable. In this paper a new, two step radiosynthesis starting from 4-benzyloxyphenyl-(2-thienyl)iodonium bromide and [^18^F]fluoride with subsequent deprotection is described, yielding n.c.a. [^18^F]fluorophenol in 34 to 36% radiochemical yield.

## 1. Introduction

The introduction of fluorine into organic molecules plays an important role in the development of new as well as in routine production of established pharmaceuticals [1]. The use of the positron emitter fluorine-18 in molecular imaging via positron emission tomography (PET) has led to a constant increase in the development of radiotracers using this radionuclide with almost ideal nuclear physical properties for PET studies. Obtaining those molecules in ^18^F-labelled form is often a great challenge. Especially, the introduction of no-carrier-added (n.c.a.) [^18^F]fluoride into an aromatic ring is difficult, especially if it is not activated for a nucleophilic substitution reaction [2]. In those cases, multi-step reactions are required to obtain the desired ^18^F-labelled radiotracer. However, starting from electron deficient small arenes like benzaldehydes, phenylketones or benzonitriles as precursors for radiofluorination, functional [^18^F]fluorobenzenes can easily be formed as intermediates and subsequently transformed by as few as possible steps to the desired radiotracer.

A further versatile primary ^18^F-labelled intermediate is n.c.a. 4-[^18^F]fluorophenol, which has proven its suitability in various radiosyntheses of biological relevant molecules or potential radiopharmaceuticals [3,4]. During the last decade, several attempts have been made to improve the synthesis of n.c.a. 4-[^18^F]fluorophenol [5,6]. The originally applied diazonium route or the recently more common radiosynthesis via Baeyer-Villiger oxidation using benzophenones as precursors require three step radiosyntheses [7] which are difficult to perform in a remotely controlled apparatus. Therefore, a more direct and convenient pathway that also minimizes any loss of radioactivity by shortening the synthesis time was examined in this work. Aryl(2-thienyl)iodonium salts have been shown as suitable ^18^F-labelling precursors even for electron rich arenes [8]. This offers the possibility of a simplified synthesis of n.c.a. 4-[^18^F]fluorophenol.

## 2. Results and Discussion

**Scheme 1:**
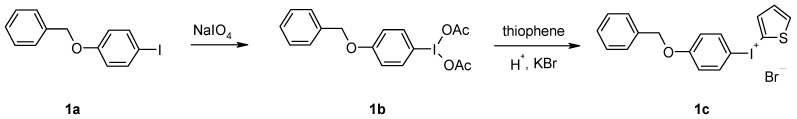
Synthesis of 4-benzyloxyphenyl-(2-thienyl)iodonium bromide (**1c**).

The need of aprotic conditions for substitution with n.c.a. [^18^F]fluoride excludes a direct ^18^F-introduction into an unprotected phenol-substituted iodonium compound. Since the benzyl protective group offers an easy and gentle way of deprotection, it was chosen for this purpose. Thus, 4-benzyloxyphenyl-(2-thienyl)iodonium bromide (**1c**) was examined as precursor. This precursor was prepared staring from 4-benzyloxy-1-iodobenzene (**1a**), which was oxidized by sodium periodate to 4-benzyloxy-1-(diacetoxyiodo)benzene (**1b**) within 2 h with a yield of 66%, as described in the literature [8]. The (diacetoxyiodo)arene and thiophene undergo an electrophilic aromatic substitution in the presence of concentrated sulphuric acid as catalyst to yield aryl(2-thienyl)iodonium hydrogen sulphate. This was subsequently converted by metathesis to its bromide salt **1c**, which was obtained in a good overall yield of 55% (Scheme 1).

Starting from dry Kryptofix^©^2.2.2/K_2_CO_3_ as anion activator system, **1c** was added to the reaction vial using DMF as solvent. The n.c.a. ^18^F-labelling reaction was carried out under the optimized conditions described recently [8]. While in that mechanistic study only the analytical radiochemical yield (RCY) of the intermediate product **1d** was reported, here the preparative radiosynthesis of 4-[^18^F]fluorophenol is further elaborated, including the debenzylation step and work-up process. Due to the deactivating effect of the *para*-benzyloxy group on nucleophilic substitution, the reaction rate was expectedly moderate. However, the ^18^F-labelling reaction led regiospecifically within 20 to 30 min to n.c.a. 4-benzyloxy-[^18^F]fluorobenzene (**[^18^F]1d**) with a RCY of 38 ± 4% as the only observed radioactive product. Before carrying out the deprotection procedure, a quick purification via Sep-Pak^©^ Plus C18 and ALOX cartridges was done. Consequently, a solvent change to methanol was easily implemented and the system was freed from polar substances and particularly from water, which had been added to enable trapping of **[^18^F]1d** on the reversed phase cartridge and to remove DMF. Methanol proved to be a suitable eluent and, moreover, also a favourable solvent in the following deprotection step.

For subsequent removal of the benzyl-moiety a reductive deprotection, applying ammonium formate and palladium black as catalyst in methanol, was elaborated. First attempts with ammonium formate and palladium on activated charcoal led to the desired n.c.a. 4-[^18^F]fluorophenol **[^18^F]1**, but difficulties were found in removing the fine charcoal particles during subsequent filtration. In addition, the system with palladium on activated charcoal resulted in a smaller RCY of 75 to 80% within 15 min at 100 °C, whereas the palladium black system yielded 90 to 95% RCY of n.c.a. 4-[^18^F]fluorophenol under the same conditions. By filtration of the reaction suspension via a LiChrolut^©^ glass column containing a PTFE frit, n.c.a. 4-[^18^F]fluorophenol was obtained in an anhydrous methanol system. A flow chart of the radiosynthesis is depicted in Table 1.

In comparison to the so far best alternative procedure for the preparation of n.c.a. 4-[^18^F]fluorophenol, described by Ludwig *et al.* [7], the route with **1c** offers two major improvements. First, the radiosynthesis is more convenient, since instead of three synthetic and two purification steps, the developed process starting from **1c** provides a less complex radiosynthesis with two synthetic and one purification steps. This offers great advantages for automation or remotely controlled radiosyntheses. Secondly, the new route saves 20 min of total synthesis time which is one of the primary concerns with ^18^F-radiosyntheses. Additionally, the resulting 4-[^18^F]fluorophenol is obtained in an anhydrous methanol system which allows for direct further radiosynthetic steps including compounds susceptible to moisture without time-consuming drying procedures. On the other hand, an unfavourable aspect is the overall lower RCY of 35 ± 1% compared to 55% with the benzophenone method. This, however, is in part compensated by the shorter synthesis time.

## 3. Experimental

### 3.1. General

All chemicals and solvents were purchased from Aldrich (Germany), Fluka (Switzerland), KMF (Germany), Acros Organics (Belgium), or Merck (Germany). They were reagent grade or better and were used without further purification. Sep-Pak^©^ Plus C18 and ALOX cartridges were obtained from Waters (USA) and LiChrolut^©^ glass columns and appropriate PTFE frits (porosity 10 μm) from Merck (Germany). Column chromatography was performed with Merck silica gel 60 (0.063–0.200 mm) and *flash*-chromatography with Fluka silica gel 60 (mesh 220–440). The eluent mixtures are given as v:v ratios. Thin-layer chromatography (TLC) was carried out with Macherey-Nagel (Germany) precoated silica gel plates (PolyGram SIL G/UV254, 40 × 80 mm) visualized under an UV-lamp (254 nm). Radio-TLC was performed on the same precoated silica gel plates. The developed radio-TL-chromatograms were measured on an Instant Imager^TM^ (Packard, USA) autoradiography system. The employed high performance liquid chromatography (HPLC) system consists of a Knauer WellChrom Mini-Star K-500 HPLC pump, a Rheodyne-Injector block 7125 and a Merck/Hitachi UV/Vis photometer L4000. HPLC was performed on a Phenomenex^©^ Luna 5 μm C18 column (3 × 250 mm) and various eluent mixtures of methanol/water or acetonitrile/water all given as v:v ratios. Radio-HPLC was performed on the same system as described for HPLC. For measurement of radioactivity the outlet of the UV/Vis detector was connected to a NaI(Tl) well-type scintillation detector (EG&G ACE MateTM). The syntheses of 4-benzyloxy-1-fluorobenzene, 4-benzyloxy-1-iodobenzene, 4-benzyloxy-1-(diacetoxy-iodo)benzene and 4-benzyloxyphenyl-(2-thienyl)iodonium bromide were performed as described in the literature [8,9] and their identity was confirmed by comparison of NMR-data and/or melting points.

### 3.2. Production of n.c.a. [^18^F]Fluoride

The production of n.c.a. [^18^F]fluoride is routinely carried out at the JSW BC 1710 Cyclotron (Forschungszentrum Jülich) by bombardment of a 96% isotopically enriched [^18^O]water target (1.3 mL, titanium body) with a 17 MeV proton beam using the ^18^O(p,n)^18^F nuclear reaction [10]. The n.c.a. [^18^F]fluoride was separated from the enriched [^18^O]water by an electrochemical cell [11]. By this procedure up to 55 GBq n.c.a. [^18^F]fluoride can be produced within 1 h at a nominal beam current of 25 μA.

### 3.3. Synthesis of n.c.a. 4-[^18^F]Fluorophenol

A 5 mL Wheaton^©^ glass vial equipped with a triangle stirring bar and a silicone septum sealed lid was connected to a vacuum line and an argon gas line, evacuated, and three times flushed with argon. From the aqueous n.c.a. [^18^F]fluoride solution 30 to 50 MBq (10–50 μL) were added to Kryptofix^©^ 2.2.2 (26 μmol) and a 1 M potassium carbonate solution (13 μL). After adding anhydrous acetonitrile (0.8 mL) the mixture was transferred through the septum into the prepared vial by a 1 mL syringe. For azeotropic distillation the vial was heated in an oil bath to 82 °C under a constant flow of argon adjusted at 750–850 mbar until no acetonitrile/water mixture had been left. This step was repeated by adding acetonitrile (1 mL × two times) which was subsequently followed by 5 min of full vacuum (~10 mbar). After argon flushing and pressure balancing the resulting dry n.c.a. [^18^F]fluoride-cryptate complex was ready for the nucleophilic ^18^F-labelling reaction.

4-Benzyloxyphenyl(2-thienyl)iodonium bromide (25 μmol) was dissolved in anhydrous DMF (1.0 mL) and added by a syringe through the silicone septum into the vial containing the dry n.c.a. [^18^F]fluoride-cryptate complex and then stirred under 1,100 mbar argon at 130 °C. At appropriate times aliquots (10 μL) were taken with a micro-syringe (Hamilton^©^) via the septum and added to cold acetonitrile (50 μL). These samples were analyzed by radio-HPLC and/or radio-TLC. After 15 to 20 min the whole reaction mixture was added into water (10 mL) using a 20 mL syringe. This solution was passed through a SepPak^©^ C18 plus cartridge preconditioned with ethanol (3 mL) and water (5 mL). The cartridge was washed with water (3 mL) and dried by an argon flow for 5 min. Before elution, the cartridge was connected to a dry SepPak^©^ ALOX cartridge. The product absorbed on the C18 phase was eluted with methanol (3 mL) and transferred through both cartridges into a new reaction vial. 4-Benzyloxy-[^18^F]fluorobenzene (**[^18^F]1d**) was analyzed by radio-HPLC [*k’* = 3.39, acetonitrile/water (40:60), flow 1.0 mL/min] and radio-TLC [R_f_ = 0.79, *n*-hexane/ether (2:1); R_f_ = 0.91, *n*-hexane/ethyl acetate (3:1)]. Ammonium formate (~5 mmol) and palladium black (~0.15 mmol) were added to the methanol solution. For debenzylation the mixture was stirred at 80 °C for 15–20 min. The whole reaction mixture was filtered through a Merck LiChrolut© glass column (65 × 10 mm) containing only a PTFE frit (porosity 10 μm) which was washed with 0.5 mL of methanol. The obtained n.c.a. 4-[^18^F]fluorophenol was analyzed by radio-TLC [R_f_ = 0.59, *n*-hexane/ethyl acetate (3:1)] and radio-HPLC ([*k*’ = 1.93] acetonitrile/water (40:60), flow 0.7 mL/min).

## 4. Conclusions

In conclusion, the reported preparation of n.c.a. 4-[^18^F]fluorophenol from 4-benzyloxyphenyl(2-thienyl)iodonium bromide (**1c**) presents an improved radiosynthesis of this primary ^18^F-labelled synthon, especially in terms of practicability of labelling and applicability of coupling chemistry in subsequently radiosynthesis steps. Thus, n.c.a. 4-[^18^F]fluorophenol was synthesised by nucleophilic substitution on 4-benzyloxyphenyl(2-thienyl)iodonium bromide and subsequent debenzylation within 40 min and an overall RCY of 34% to 36%. This new approach towards the versatile ^18^F-labelling synthon 4-[^18^F]fluorophenol offers a short and very convenient two synthetic and one purification step procedure. In addition, the final 4-[^18^F]fluorophenol is obtained in an anhydrous methanol solution which facilitates a broad variety of following coupling steps, even with moisture sensitive molecules. Preparation of the useful 4-[^18^F]Fluorophenol via the 4-benzyloxyphenyl(2-thienyl)iodonium bromide precursor thus requires a far less complex radiosynthesis which enables an easy transfer to a remotely controlled synthesis device.

## Figures and Tables

**Table 1:** Synthetic sequence of the two step radiosynthesis of n.c.a 4-[^18^F]fluorophenol.

Time (min)	Structures	Compounds & Conditions	RCY (%)
0 20 40	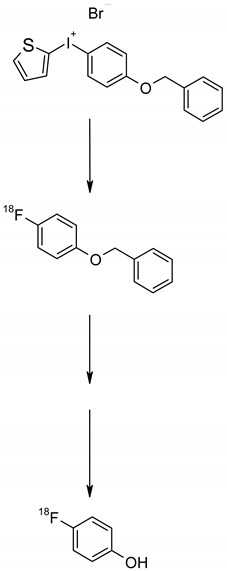	**1c** [K⊂2.2.2.]_2_CO_3_, c(**1c**) = 25 mmol/L, DMF, 130 °C, 1100 mbar Argon **[^18^F]1d** cartridge purification eluent: MeOH (2 mL) HCOONH_4_; Pd (black) MeOH; 100 °C; filtration **4-[^18^F]Fluorophenol**	34–38 34–36 (90–95) *

* RCY related to deprotection step.
